# The Action of Certain Drugs on the Heart

**Published:** 1911-03-11

**Authors:** J. M. Fortescue-Brickdale

**Affiliations:** Physician to Clifton College; Assistant Physician to the Royal Infirmary, Bristol.


### Hospital Clinics.

### THE ACTION OF CERTAIN DRUGS ON THE HEART,

### WITH NOTES ON THEIR PREPARATIONS AND PRACTICAL APPLICATION.

By J. M. FORTESCUE-BRICKDALE, M.A., M.D. Oxon., Physician to Clifton College; Assistant Physician to the Royal Infirmary, Bristol.

It is now very generally recognised, I think, that in order to combat disease in an effective manner we must make a careful study not only of the powers and characteristics of our enemy, but also of the weapons with which we intend to carry out our attack. Among these weapons drugs have from the earliest period in the history of medicine occupied an important position; it is true that during the latter half of the last century there was a tendency to minimise the value of drug treatment, and to rely to a great extent on other factors, such as rest, diet, and climatic influences, in securing an amelioration in morbid conditions; but more recently the view has been gaining ground that the careful and judicious use of drugs is capable of influencing the result to a more considerable extent than had hitherto been imagined, and consequently that increased attention should be given to this subject both by students and by practitioners. The scientific study of pharmacology has, moreover, made great advances during the last decade both in extent and accuracy of method, and has, moreover, become more definitely linked with the practical business of therapeutics. It is with the object of setting forth some of the recent conclusions arrived at by workers in one portion of this domain that I have selected as a subject for this lecture "The Action of Certain Drugs on the Heart." The behaviour of the heart under treatment in various diseased conditions illustrates very well the remarks with which I have prefaced this lecture.

The principal cardiac drug is still, no doubt, digitalis in some form or other. This drug was, as is well known, introduced into official medicine by Witherington in 1775; its chemistry and pharmacology were, to a great extent, worked out by Schmietteberg, Fraser, and others in the middle of the last century, and its physiological action has recently been more minutely studied by means of improved instruments for recording the action of the heart. Nevertheless, there has been a distinct tendency to assume, until recently at any rate, that the drug treatment of cardiac affections was comparatively unimportant; that the effects obtained were unreliable, and that as a rule general measures were all that was necessary in dealing with such cases. At the same time there has been I fear a habit set up of treating abnormal cardiac *sounds* with small doses of digitalis in a manner regardless alike of the action of the drug, the physical indications for its use, or the pharmaceutical efficiency of the preparation and dose. Rational drug therapy in cardiac diseases has no doubt been much hindered by the difficulties experienced in obtaining preparations of a constant strength, and by the ignorance which still exists as to the relative values of the many active principles involved, whilst the very marked benefit of other methods of treatment in many cases has led to neglect of this somewhat confusing branch of therapeutics. I propose in this lecture to endeavour to clear up certain points in the consideration of this subject as far as our present knowledge will allow, and for that purpose I shall divide my remarks into three sections. The first will deal with the various preparations of digitalis and its allies now on the market; in the second I shall endeavour to show you the present state of our knowledge as to the action of these bodies on the heart; the third section will be devoted to the therapeutic indications for cardiac drugs and to the important questions of dosage and method of administration. I shall throughout avoid as far as possible purely theoretical and experimental details, and if in the end the conclusions we arrive at are consonant with those you have obtained in practice, I hope that by pointing out to you, however briefly and imperfectly, the basis on which those conclusions stand, I may help you to make further advances in practice, and a freer and more confident use of the knowledge which experience has already placed at your disposal.

#### I. (*a*) Preparations of Digitalis.

There are, as you know, two classes of compounds prepared from digitalis. First of all there are drugs, like the tincture or infusion, which contain a mixture of glucosides; and next there are the so-called active principles in pure form. The objection usually made to the first class of compound is that it is uncertain in action. As you know, the digitalis plant varies very much in its content of glucosides, and besides this the preparations keep badly. The active principles being glucosides are liable to split up under the action of ferments, and perhaps under other conditions also, into a sugar or carbohydrate and another body: the carbohydrate, of course, is inactive, and it is a general rule, to which there are but few exceptions, that the other substance derived from the decomposition of a glucoside is less active physiologically than the glucoside itself. In order to obtain a more reliable drug, the principle glucoside, digitoxin, has been assayed chemically. The usual process by which this is done, which was originally devised in 1897, is not very accurate, and besides this it has repeatedly been found that the activity of a digitalis preparation is not directly proportional to its digitoxin content. Various methods have, therefore, been devised of estimating the physiological activity of samples of digitalis by experiments on animals, either by determining the minimal lethal dose for some small animal, or by measuring the effect on the blood-pressure of a large animal, or by experiments on the frog's heart. Now it has been found that the same specimen of digitalis, tested by these three methods, gives variable results, so that even the physiological test is not altogether in a satisfactory position. Probably (if carefully conducted) experiments on the frog's heart give the most valuable results, but, as I shall point out to you later, they do not show us the effect on the central nervous system to which the drug when used in practice owes a portion at least of its pharmacological action.

Several special preparations made from the entire leaves are on the market, which I will now shortly enumerate.

*Digitalisatum* (Bürger) is a watery extract containing also a little alcohol, 1 grain of which corresponds to 1 gram of the dried leaves, or 0.7 gram of pure digitoxin. The dose is sixteen to twenty drops. This preparation is tested physiologically and is said to keep well.

*Digitalis Dialysatum* (Golaz) is a somewhat similar preparation, which is obtained by dialysation of the juice of fresh leaves. It is also standardised physiologically. The dose is twenty drops.

*Digipuratum* (Knoll) is a dry extract obtained in the form of a powder or as tablets, each of which is equal to $1\frac{1}{2}$ grain of the leaves. It is physiologically standardised and is stable. The dose recommended is four tablets during the first twenty-four hours, the amount being diminished on succeeding days as the effect of the drug becomes apparent. Or, when larger doses are necessary, as much as five tablets may be given daily for two or three days, and the dose then diminished.

*Digitalone* is a watery extract to which chloreboræ is added. It is said to keep well, but this has not always been found to be the case. It is about half as strong as the pharmacopœial tincture.

Of the principal preparations of the glucosides themselves several are known as digitalin, but this does not imply that the active constituent is the glucoside of that name.

*Nativelle's Digitalin* is a digitoxin preparation, the dose of which is one six-hundredth to one-sixtieth of a grain. The glucoside is insoluble in water and is prepared in "granules."

*Merck's Digitoxin* is a crystalline powder soluble in alcohol and chloroform, but not in water. It is sold in compressed tablets or in powder; the tablets contain $\frac{1}{4}$ milligram each, and two are usually required daily in ordinarily severe cases. The drug may also be dissolved in alcohol and made up with chloroform-water. Similar doses may in severe cases be injected intravenously.

*Kiliani's Digitalinum Verum* (the average dose of which is $\frac{1}{100}$ grain) is the glucoside known by that name, which is soluble in alcohol and mainly obtained from the seeds and not the leaves of the plant. It is not so active as digitoxin.

*Homelle and Quevenne's* digitalin is an amorphous powder, the composition of which is uncertain. It cannot, therefore, be said to present any advantages.

*German digitalin* is a mixture of glucosides, containing no digitoxin, and is mainly used for hypodermic injection owing to its solubility in water. The dose is $\frac{1}{60}$ to $\frac{1}{12}$ of a grain.

*Digalen* is a somewhat recent preparation which is stated by its discoverer, Cloëtta, to be a soluble amorphous form of digitoxin. Others state that it is merely an impure digitalein; it is generally intended for intravenous use, but cannot be given hypodermically as it is too irritating. The dose by the mouth varies from 17 mimims to 1 drachm thrice daily, according to the severity of the case.

The effect must be carefully watched, as it is probably cumulative to some extent. The dose, as for other digitalis preparations, is gradually decreased day by day in accordance with the symptoms observed. One half to 1 c.c. may be given intravenously in twenty-four hours, if this method of administration is preferred. With regard to the comparative efficiency of these preparations the general opinion of clinicians in this country at any rate is that a good tincture or infusion of digitalis acts better than the isolated glucosides. In all cases it is well to use a preparation which has been physiologically standardised, and to obtain it from a manufacturing house of high reputation. Some of the older authorities were in the habit of using the dried powdered leaves, and a recent observer has shown that the infusion is 20 per cent. weaker than the dry preparation from which it is made, and is similarly poorer in the chemical bases. Practically the powder must be given in cachets, and, like most solid drugs, it is absorbed more slowly. The addition of alcohol may enhance the physiological effect, but as a rule digitalis is best given alone, or with a little sodium bicarbonate or liquor bismuthi, which may prevent some of the digestive disturbances. Acids, acid salts (such as the perchloride of iron), and potassium salts should not be combined in the same mixture with digitalis.

(*b*) Preparations of Strophanthus.

Of these, as in the case of digitalis, there are two classes, those containing all the active principles and those purporting to represent pure glucosides. Several varieties of the strophanthus plant yield glucosides which to a certain extent have a similar action, but marked differences also exist. In contradistinction to the preparations of digitalis, the pure glucosides are generally thought to give better results than the tinctures, and it is on these, therefore, that I shall now offer a few remarks. The glucosides are generally named strophanthin, but at least three distinct bodies are so called. *Crystalline strophanthin*, also called *strophanthin g.*, because it is derived from the variety *strophanthus gratus*, is the most toxic. It is apparently identical with the African arrow-poison *ouabain*, which in an amorphous form has been employed as a cardiac drug, as it may be injected hypodermically. The British Pharmaceutical Codex calls *strophanthin g.* pseudo-strophanthin. It is soluble in alcohol or normal saline, the dose by the mouth is $\frac{1}{5}$ to $\frac{1}{2}$ grain in twenty-four hours, the maximum single dose being $\frac{1}{12}$ grain. Intravenously or intramuscularly $\frac{1}{120}$ to $\frac{1}{60}$ grain may be given once in twenty-four hours.

*Amorphous strophanthin* (Merck) is a less powerful body. It is intended for intravenous injection in doses of $\frac{1}{120}$ to $\frac{1}{60}$ grain; it is too irritant for intramuscular injection.

*Amorphous strophanthin* (Boehringer) is still less active, and may be given intravenously up to $\frac{1}{30}$ grain, or intramuscularly up to $\frac{1}{20}$ grain in twenty-four hours. By the mouth a single dose should not exceed $\frac{1}{60}$ grain, while four times that amount may in severe cases be given in twenty-four hours.

By the mouth all preparations of strophanthin give less certain results owing to rapidity of excretion; while, for the different varieties, the considerable differences in the rate and completeness of absorption which have been noted render this method of administration inadvisable when a rapid and certain effect is urgently required.

(*c*) The preparations of squill, camphor, convallaria, and other drugs which act on the heart in a manner more or less similar to that of digitalis do not need special mention. At present precise information regarding most of them is scanty.

II. In this section the most convenient method of setting the known facts before you will be to consider first the action of digitalis, and then to note in what respects the other cardiac drugs differ from it or resemble it.

Digitalis has a double action on the mammalian heart—namely, a stimulant action on the vagus centre in the medulla, and also a stimulant action on the cardiac muscle itself. To the first of these is due the slowing of the rate which is seen after the administration of digitalis, and also, according to many, the irregular rhythm which is induced or accentuated by small doses of the drug. By its action on the heart-muscle it increases the strength of the contractions, and possibly improves the tone of the ventricular walls; it may also increase the excitability of the heart-muscle in large doses, and so set up irregularity of rhythm. The action on conductivity is possibly a mixed one, partly central and partly muscular; it results in a lowering of the conductivity, and may produce partial or complete "heart block." Besides its direct effect on the heart digitalis constricts the peripheral vessels, but in practice observers do not always find that the blood-pressure is raised even when large doses are given. The splanchnic vessels are thought by some authorities to be the most markedly affected, though some of the digitalis glucosides undoubtedly affect the peripheral vessels in the limbs.

As compared with digitalis, strophanthus acts far more powerfully on the amphibian heart, but in man the same result is not obtained, owing to malabsorption and other factors which have been already noted. Its action as a vaso-constrictor is also much less. It is not so likely to set up vomiting, the usual digestive disturbance after its use being diarrhœa.

As regards squill there is some uncertainty. It appears to have a greater vaso-constrictor effect than digitalis and to be a more powerful diuretic. It resembles strophanthus in giving rise to diarrhœa rather than to vomiting; although some observers credit it with a more powerful action on the heart than digitalis, it is generally held that it is not so active, though at the same time probably not so toxic. Camphor is employed by some Continental clinicians as a cardiac stimulant; how it differs from or resembles digitalis it does not at present seem possible to state. Cactus, though it has been praised in certain quarters, is an unreliable drug, and the commercial varieties are often completely inactive. Helleborein is said to have less action on the vagus than other bodies in this group, but at present the clinical experience as to its value is scanty. Of the pharmacology of the other drugs such as convallaria, apocynum, and crategus there is at present but little positive knowledge.

III. The large amount of clinical experience in the use of digitalis which has accumulated since its introduction has on the whole fully confirmed the observation of Witherington that "if the pulse be feeble and intermitting, the countenance pale, the lips livid, the skin cold, the swollen belly soft and fluctuating, or the anasarcous limbs pitting readily under the pressure of the finger, we may expect the diuretic effects to follow in a kindly manner." These are, in fact, cases of valvular disease, usually rheumatic in origin, with marked signs of backward pressure and low arterial tension. As regards dosage, it is usually thought that, at any rate at first, full doses should be given; in fact, it has been shown that the rapidity of the therapeutic effect is proportional to the size and frequency of the dose. Fifteen minims of the tincture every four hours is necessary in bad cases, the dose to be reduced when an improvement in the symptoms appears, or when headache, nausea, vomiting, giddiness, or distressing cardiac action sets in. Opinions are divided as to the danger of inducing digestive disturbances by digitalis, but it seems certain that the vomiting may sometimes be very intractable and seriously interfere with the patient's chances of recovery. Where the heart symptoms are less urgent smaller doses, such as 15 m., thrice daily, may be given with advantage. To control the adverse action on the stomach the doses may be given in iced water directly after food. When there is extreme hypertrophy of the left ventricle with mitral regurgitation the action of digitalis is sometimes too marked, causing much reflux into the auricle, and a dilatation of the comparatively weak right side of the heart. Also when the muscular wall is excessively weakened the contraction of the peripheral arteries may be more than the enfeebled heart can meet, and in these cases the drug does not do good. Where there is persistent vomiting or marked digestive disturbance digitalis will also be contra-indicated. Aortic incompetence when setting up symptoms of backward pressure may be treated by digitalis with advantage, provided that the myocardium is fairly healthy, and that the patient is not suffering from marked arterio-sclerosis. When the now well-known phenomenon of "extra systole" is present, it is probable that digitalis has but little effect on its frequency. Small doses have been found in some cases to lessen their occurrence, though larger ones may increase them or produce them. In the heart failure of acute fevers digitalis often fails. It is, however, especially in cases of heart block that digitalis is to be withheld, as by diminishing the conductivity it merely renders this condition more marked. In aortic stenosis it also fails to do good. Strophanthus is very generally considered of less value than digitalis, though it may perhaps be given more freely where pulse-tension is raised. Large doses are generally necessary if the drug is given by the mouth, up to a drachm or a drachm and a half in twenty-four hours. Recently the use of tincture of squills has been revived, but the evidence that it possesses any real advantage over digitalis as a cardiac drug is not very strong, although it is more easily tolerated by the stomach. The effective dose is the same as that of tincture of strophanthus.

I will now pass on to consider the intravenous administration of some of the digitalis and strophanthus glucosides. This procedure has been recommended in order to produce a rapid effect in severe cases, and apparently the most suitable drug to employ is some preparation of strophanthin. In severe cases one milligram of Merck's strophanthin may be injected, and, if necessary, repeated within twenty-four hours. Usually only one injection a day is required. In less severe cases half the dose is sufficient. The action is rapid and does not differ from that of any other digitalis drug, nor are the toxic effects different. It is only suitable for cases of cardiac failure with arrhythenia, low-tension pulse, and backward pressure symptoms. In cases with well-marked nephritis the action is but slight. There is unfortunately a certain number of cases in which death has occurred immediately after an injection of strophanthin, which has obtained for this treatment a somewhat unenviable reputation. Rigors and fever have also been observed after the injection of 1 milligram of amorphous strophanthin. To avoid such accidents the following precautions must be observed: the strophanthin should not be given where the patient has recently been taking digitalis; the dose should not be too large or two frequently repeated; it should not be employed in cases of advanced arterio-sclerosis or where embolism has occurred or appears likely.

Lastly I should like to say a few words about the less commonly used cardiac drugs, which may be considered to be still in the experimental stage. Helleborein has been already alluded to; it has the disadvantage of acting as an intestinal irritant. Adonidin acts somewhat similarly to strophanthin in that it does not contract the coronary arteries. Apocynum is an irritant to the stomach and also the kidneys; it owes its efficacy to a somewhat insoluble bitter substance, cynotoxin. The so-called glucoside aposynin is inactive. Crategus has been recommended in cases of cardiac failure with atheroma, and also in the palpitation of functional nervous disorders. Convallaria has also been prescribed for the latter class of case. Cactus I have already alluded to as being an uncertain and unreliable drug. It has been recommended in exactly that class of case in which it is practically impossible to exclude other factors in the production of the therapeutic effect, such as rest, diet, and, above all, suggestion. There appear to be few unpleasant symptoms attending its administration, and, indeed, it has been shown that many commercial specimens of the drug are inert.

The upshot of the whole matter seems to be that in cases of cardiac failure unaccompanied by symptoms of "heart block" and in which there is no marked digestive disturbance or very high-pulse tension, a good preparation of the official tincture of digitalis in sufficient doses is the most likely to benefit the patient; other drugs may be tried in less urgent cases, but many of them are still in the experimental stage, and of the better-known ones it is doubtful whether they show any marked advantages over the tincture of digitalis, either as to efficiency or absence of undesirable side-effects.

